# Cryoablation and immunity in non-small cell lung cancer: a new era of cryo-immunotherapy

**DOI:** 10.3389/fimmu.2023.1203539

**Published:** 2023-08-21

**Authors:** Antonio Velez, Andrew DeMaio, Daniel Sterman

**Affiliations:** Pulmonary Oncology Research Team (New York University (NYU) PORT), Section of Interventional Pulmonology, Division of Pulmonary, Critical Care and Sleep Medicine, NYU Grossman School of Medicine, NYU Langone Health, New York, NY, United States

**Keywords:** cryoablation, cryotherapy, lung cancer, immune response, bronchoscopy

## Abstract

Despite remarkable advances in tumor response and patient survival in the past decade, systemic immunotherapies for lung cancer result in an objective response in only around half of patients treated. On the basis of this limitation, combination strategies are being investigated to improve response rates. Cryoablation has been proposed as one such technique to induce immunogenic cell death and synergize with systemic immunotherapies, including immune checkpoint inhibitors. Cryoablation has been traditionally delivered percutaneously with imaging guidance although recent technological advances allow for bronchoscopic delivery. Herein, we review the pre-clinical and clinical evidence for the use of cryoablation in non-small cell lung cancer and potential induction of anti-tumor immunity. We highlight ongoing studies involving this approach and propose areas of future investigation.

## 1 Introduction

Immune checkpoint inhibitors, including antibodies to programmed death cell death protein 1 (PD-1) and its ligand PD-L1 have revolutionized the treatment of patients with non-small cell lung cancer over the past decade. However, response rates to these therapies remain suboptimal even when the tumor demonstrates significant PD-L1 expression. Thus, combination immunotherapy approaches which use immune checkpoint inhibitors in combination with other therapeutic interventions have been proposed with the goal of improving overall clinical responses.

The cancer-immunity cycle has been proposed as a framework of seven steps required for the killing of cancer cells by the immune system (1). Specifically, the steps include: 1) release of tumor associated antigens and immunomodulatory danger signals, (2) cancer antigen presentation, (3) priming and activation of immune cells ,(4) trafficking and (5) infiltration of T cells into tumors, (6) recognition of cancer cells by T cells, and (7) immune-mediated cell death leading to further tumor antigen and danger signal release.. Each of these steps has been targeted by investigators for its potential to combine with current immunotherapies to improve response rates.

Release of cancer cell antigens is pivotal to generation of an anti-tumor immune response and may be augmented in several ways. For example, local treatments (tumor ablation, intratumoral injection, radiotherapy) and systemic therapies have been proposed as strategies to increase tumor antigen release and presentation to the immune system. Additionally, an increasing body of evidence suggests that immunogenic cell death may sensitize patients to PD-1/PD-L1 blockade (2). One particular local therapy, cryoablation, has been studied for its ability to generate an anti-tumor immune response and may have advantages over several of the other proposed methods.

In this review, we will discuss the proposed immunologic mechanisms of cryoablation and highlight pre-clinical and clinical investigations using cryoablation as part of a combination immunotherapy strategy.

## 2 Mechanisms of cryoablation

Cryoablation is a thermal ablation technique in which cold temperature is used to induce tissue injury. Although the therapeutic properties of cold have been utilized for centuries, the modern era of cryosurgery began in the 1960s when the neurosurgeon Dr. Irving Cooper developed a modern cryosurgical system using a probe for treatment of brain tumors and movement disorders (3). Since then, cryoablation has been utilized for local treatment of several malignancies including liver, kidney, breast, prostate, lung and bone (4).

To achieve low temperatures at the tip of the probe, a compressed gas (i.e., cryogen) is passed through a valve where it rapidly expands, resulting in cooling *via* the Joule-Thomson effect. A variety of cryogens are utilized clinically including liquid nitrogen, argon, and carbon dioxide, which produce a range of cold temperatures at the tip of the probe within seconds of application (5). By direct application of this temperature to target tissues, tissue injury and cell death are locally induced. It is postulated that this tissue injury in turn leads to antigen presentation and the development of an antitumor immune response both locally and at distant sites (7). This will be further discussed in Section 6, Immune Response to Cryoablation.

Cryoablation results in cell death *via* an immediate direct cell injury, as well as delayed effects due to vascular thrombosis and potentially immune-mediated pathways (5). First, the direct application of cold damages target tissues and causes crystallization of extracellular water, leading to a hyperosmotic extracellular space resulting in osmotic injury to cells. With application of colder temperatures, direct intracellular crystallization impairs function of membranes and organelles, leading to cellular dysfunction and death. Following this direct effect, microvascular stasis occurs resulting in thrombosis, local ischemia and additional cell injury. While cells not subjected to lethal injury may be able to recover, temperatures of -20°C to -50°C may induce complete necrosis, which notably depends on the nature of the frozen tissue (6). For example, neoplastic cells may be more resistant to lethality from cold temperature than benign tissues. The effects of cold injury depend on many factors, including the tissue frozen, rapidity and duration of freeze and thaw cycles, number of freeze-thaw cycles (4, 5).

The optimal “ablative dose” is not known and many have investigated different protocols to optimize cryosurgical techniques (8). Thus, several authors have proposed a target temperature of -40 C for at least one minute in order to achieve a lethal effect in neoplastic tissues (4). In general, a rapid cooling phase, followed by a slow thaw phase, and repeated freeze-thaw cycles increase the amount of cell death with cryoablation (4). In a model of pulmonary cryoablation, three freeze-thaw cycles were suggested to be superior to two cycles for overall efficiency and visualization of the cryoablation zone. In particular, the first freeze-thaw cycle may improve thermal conductivity of lung tissue and tumor to amplify the freeze effect during subsequent cycles (9). Further investigation is needed to determine optimal ablation protocols.

## 3 Cryoablation versus other thermal ablative modalities

In comparison with other modalities of image-guided thermal ablation such as radiofrequency ablation (RFA) and microwave ablation (MWA), cryoablation offers several possible advantages as part of an immune stimulating strategy. Certain tissues have an intrinsic cryo-resistance due to low water content (including cartilage, a component of the bronchial wall) which may minimize damage to normal tissues surrounding a tumor (10). Thus, cryoablation is the preferred image-guided ablation technique for tumors near the large airways, diaphragm or pleura (11). A meta-analysis comparing thermal ablative therapies for lung cancer reported no statistically significant differences in adverse reactions between cryoablation, radiofrequency ablation (RFA), and microwave ablation (MWA) (12). Additionally, cryoablation may be less painful that other thermal modalities as cold temperatures have an analgesic effect on tissues (13). Finally, cryoablation differs from RFA and MWA in that it does not produce extreme heat which is known to denature tumor antigens, and therefore may better preserve tumor antigens for processing by the immune system.

## 4 Current uses of cryoablation in thoracic malignancies

### 4.1 Percutaneous cryoablation

Percutaneous cryoablation is an image-guided thermal ablation technique which is used for the treatment of early-stage peripheral lung cancer, multiple primary lung cancers, or metastatic disease to the lung, typically in medically inoperable patients. Additionally, image-guided ablation has been used for recurrent disease or symptomatic tumor invasion of the chest wall. The major benefits over surgery include sparing of lung parenchyma, with pulmonary function testing post tumor cryoablation revealing minimal to no sustained reduction in FEV1 or DLCO (14). Several societal guidelines support its use as an alternative to radiotherapy in patients who are “high risk,” defined as medically inoperable or borderline operable (14, 15).

A number of studies over the past decade have revealed safety and efficacy of percutaneous cryoablation (16–23). Pneumothorax is the most common complication, requiring chest tube placement in around 20% of patients (11, 24). The incidence of severe adverse events (defined as grade 3 or higher according to the National Cancer Institute Common Terminology Criteria for Adverse Events) is low, occurring in around 6% of patients (18, 20, 21). Rarely, bronchopleural fistula or hemorrhage may occur.

### 4.2 Bronchoscopic cryoablation

Bronchoscopic cryoablation is a technique used for destruction of tissue within the airways. Most commonly, it is applied for palliative treatment of malignant airway obstruction (25, 26). Bronchoscopic cryoablation has traditionally been applied by direct application of a cryoprobe *via* several freeze-thaw cycles. Recently, spray cryotherapy has been investigated as another delivery method (27). Bronchoscopic cryoablation has also been utilized for the curative-intent treatment of endobronchial malignancy in patients not eligible for surgical resection (27). This technique has primarily been utilized in superficial malignancies involving the central airways including carcinoma *in situ* and carcinoid tumors with demonstrated safety and clinical efficacy (28–30).

Transbronchial cryoablation for therapeutic intervention with curative intent of inoperable peripheral lung tumors is being investigated, but remains limited to research settings only (31–34). The techniques of transbronchial cryobiopsy and cryo-recanalization are not discussed here as the technique does not leave sufficient frozen tissue *in situ* for processing by the immune system and is thus postulated not to have the same immunomodulatory effect.

## 5 Delivery approaches for cryoablation as a combination immunotherapy

### 5.1 Percutaneous

There is accumulating clinical evidence for the use of percutaneous cryoablation for non-small cell lung cancer, although the procedure is currently performed only at specialized centers in the United States. For thoracic indications, imaging guidance is typically performed with computed tomography allowing precise lesion targeting. Percutaneous probes commonly use argon for cooling and helium for rewarming, giving them the ability to reach temperatures of as low as -140°C at the probe tip (35). Commonly used probe sizes for lung ablation range from 1.5 to 2.5 mm in diameter, and a single probe has the capacity to create an ablation zone (defined by a measured -20°C isotherm) of up to 40 mm in largest dimension depending on probe size (13). Cryoablation has the benefit of improved visualization of the ablation zone when compared to other thermal ablative methods (17). This allows for placement of several cryoprobes to increase the cryoablation zone to completely encapsulate a lesion.

Negative aspects of the percutaneous approach include breach of the visceral pleura, which is associated with a risk of complications. In fact, pneumothorax may occur in up to 50% of percutaneous cryoablation procedures, requiring chest tube placement in about 20% (11, 24). Some have suggested that tract embolization with gelatin sponge slurry may reduce the incidence of pneumothorax after radiofrequency ablation of lung tumors (36), but the quality of evidence is limited and we could not find any reports of its use with percutaneous cryoablation. Thus, alternative approaches to cryoablation of lung tumors have been sought which do not cross the visceral pleura.

### 5.2 Bronchoscopic

Bronchoscopic cryoablation has been postulated as a potential way to reduce risks of cryoablation for lung tumors as the cryoprobe can be directed within the airways and does not cross the visceral pleura. With improving bronchoscopic navigation technologies and confirmation of probe positioning with cone-beam CT, bronchoscopic cryoablation may soon be feasible. Bronchoscopic cryoablation may also allow for access to multiple intrathoracic thoracic lesions, including the future potential for treatment of nodal metastasis.

A disadvantage of bronchoscopic cryoablation is the challenge of encapsulating the entire lesion within the ablation zone, as cryoprobes delivered through an airway are not as easily positioned as percutaneous probes under CT guidance. Additionally, currently available flexible cryoprobes have inferior ablation zones compared to rigid percutaneous probes. Rigid probes may allow for shortening of the thawing phase, therefore shortening procedure time, by a mechanism that prevents decompressed gas from returning into the cryoprobe. On the other hand, longer thawing time may assist in cellular injury, which could be enhanced with the flexible cryoprobe (27). Although rigid cryoprobes can be used through a rigid bronchoscope, these are solely for endoluminal lesions and the reach of these probes is limited mostly to central airways and proximal lower lobes.

Preclinical studies using a cryoprobe for bronchoscopic delivery of cryoablation have shown feasibility. *Kohno et al.* demonstrated the feasibility of transbronchial cryoablation in a porcine model using a rigid cryoprobe (31). Temperature measurements during the cryoablation and histologic evaluation of the ablation zone after animal sacrifice were performed. Although the rigid cryoprobe is unable to reach many peripheral lung lesions, its technical feasibility and lack of serious adverse effects in the ablation zone were demonstrated.

Subsequently, in an *ex vivo* model utilizing an explanted porcine lung, *Zheng et al.* used a prototype 2.3 mm flexible cryoprobe through a bronchoscope to evaluate feasibility of transbronchial cryoablation (37). This specially designed cryoprobe reaches a temperature of nearly -160 C at the tip using liquid nitrogen as the cryogen. In this preclinical study, the probe tip created an ice ball of up to 3.5 cm in diameter in porcine lung and the target temperature was reached close to the cryoprobe. Specifically, a threshold temperature of -20°C or less was reached within 12 mm from the cryoprobe. A further study by this group demonstrated the feasibility of transbronchial cryoablation in an *in vivo* porcine model (33). Another research group used a commercially available 2.4 mm flexible bronchoscopic cryoprobe (ERBECRYO2; Erbe Elektromedizin GmbH, Tubingen, Germany) to ablate a gelatin model of lung tissue (34). Although the pre-specified ablation temperature (-20°C) was reached after 15 minutes with a double freeze protocol, the ablation zone was small due to the limited ablation effect of this carbon dioxide-based probe, which can only reach temperatures as low as -79°C. Thus, further work is needed to develop bronchoscopic devices which may provide an enhanced ablative effect.

An additional potential benefit of the bronchoscopic cryoablation is the ability to combine with standard of care diagnostic or therapeutic bronchoscopy procedures. Our institution is currently recruiting for a safety and feasibility trial of bronchoscopic cryo-immunotherapy (BCI) for non-small cell lung cancer (ClinicalTrials.gov identifier NCT04049474). This phase I study is designed to evaluate the safety, feasibility and preliminary systemic immune response to dose-escalated cryoablation of a peripheral lung tumor delivered at the time of diagnostic or therapeutic bronchoscopy for known or suspected advanced non-small cell lung cancer. Dosing in this trial is determined by freeze time with standardization of the optimal freeze temperature achievable with standard flexible cryoprobes. This study utilizes a radial ultrasound probe (UM-S20-17S; Olympus, Tokyo, Japan) *via* a guide sheath to confirm as close as possible to a concentric view of the peripheral lesion prior to insertion of the cryoprobe under fluoroscopic guidance. The maximal freezing time in this current study design is 30 seconds per centimeter of tumor, which is a far shorter duration than was achieved in the porcine studies. It is possible that longer freeze durations will be necessary for optimal tumor cell killing and antigen presentation. Additionally, the protocol allows for usage of robotic bronchoscopy for localization of the peripheral lesion and to facilitate cryoablation of target tumors using the 1.1 mm diameter flexible cryoprobe (ERBECRYO2; Erbe Elektromedizin GmbH, Tubingen, Germany). The robotic bronchoscope’s working channel allows for passage of the 1.1 mm probe without the need for insertion of a guide sheath. The shape-sensing technology implemented in the robotic bronchoscope (Ion Endoluminal System; Intuitive Surgical, Sunnyvale, California), allows for stable positioning of the cryoprobe within the lesion despite the device exchanges of the biospy forceps and the radial ultrasound probe beforehand.

There have been several studies published in the medical literature of the use of robotic bronchoscopy for cryobiopsy of peripheral lung lesions (38, 39), but no published studies have demonstrated the use of the 1.1 mm flexible cryoprobe for therapeutic purposes in patients with lung cancer. In addition, the use of advanced fluoroscopy and/or cone-beam computed tomography allows for confirmation of the presence of the cryoprobe within the target lesion to optimize the cryoablation procedure. In the current protocol, the immune response to BCI is determined solely by assessment of peripheral blood samples taken just prior to the study procedure and at 7 and 14 days post-procedure. In future iterations of BCI, we aim to obtain repeat tumor biopsies and endobronchial ultrasound (EBUS)-guided nodal aspirates after several weeks to be able to better discern the effects of BCI on the tumor and draining lymph node immune microenvironments.

Recently, several studies have investigated the use of the EBUS-guided cryobiopsy of mediastinal and hilar lymph nodes to obtain larger biopsy specimens for improved diagnosis of disorders such as lymphoma and sarcoidosis (40, 41). The procedure involves first accessing the target node using the linear EBUS bronchoscope and large biopsy needle or needle-knife. Subsequently, the 1.1 mm cryoprobe is advanced through the working channel and into the lymph node under EBUS guidance with freezing and cryoadhesion of nodal tissue. In the future, there may be an immunologic role for therapeutic cryoablation of tumor-involved lymph nodes. Induction of cell necrosis *via* cryoablation within the nodal environment may facilitate enhanced tumor antigen presentation to naïve CD8 T cells and increased production of tumor-directed cytotoxic T cells which can then enter the systemic circulation. Furthermore, the potential for nodal cryoablation to work in synergy with immune checkpoint inhibitors and combat immune checkpoint inhibitor resistance was explored in a published case of aortocaval lymph nodal metastasis from immunotherapy-resistant non-small cell lung cancer which was treated successfully with percutaneous cryoablation while continuing the immune checkpoint inhibitor (42). It remains to be studied whether such effects are reproducible, and whether they can be extrapolated to nodal ablation *via* EBUS.

## 6 Immune response to cryoablation

Studies dating back to the 1960s have suggested a potential immune response to cryoablation. Specifically, it was initially recognized that cryoablation caused formation of auto-antibodies against the frozen tissues (43). Characterization of the immune response was limited by the laboratory techniques at the time. However, there were several case reports in the 1970s of regression of distant lesions (“abscopal” effect) following cryoablation of prostate tissue (44, 45). Since then, technologies for assessment of the immune response have improved and allowed for further investigation into potential mechanisms of immune response to cryoablation, although the understanding remains incomplete.

**Figure 1:**
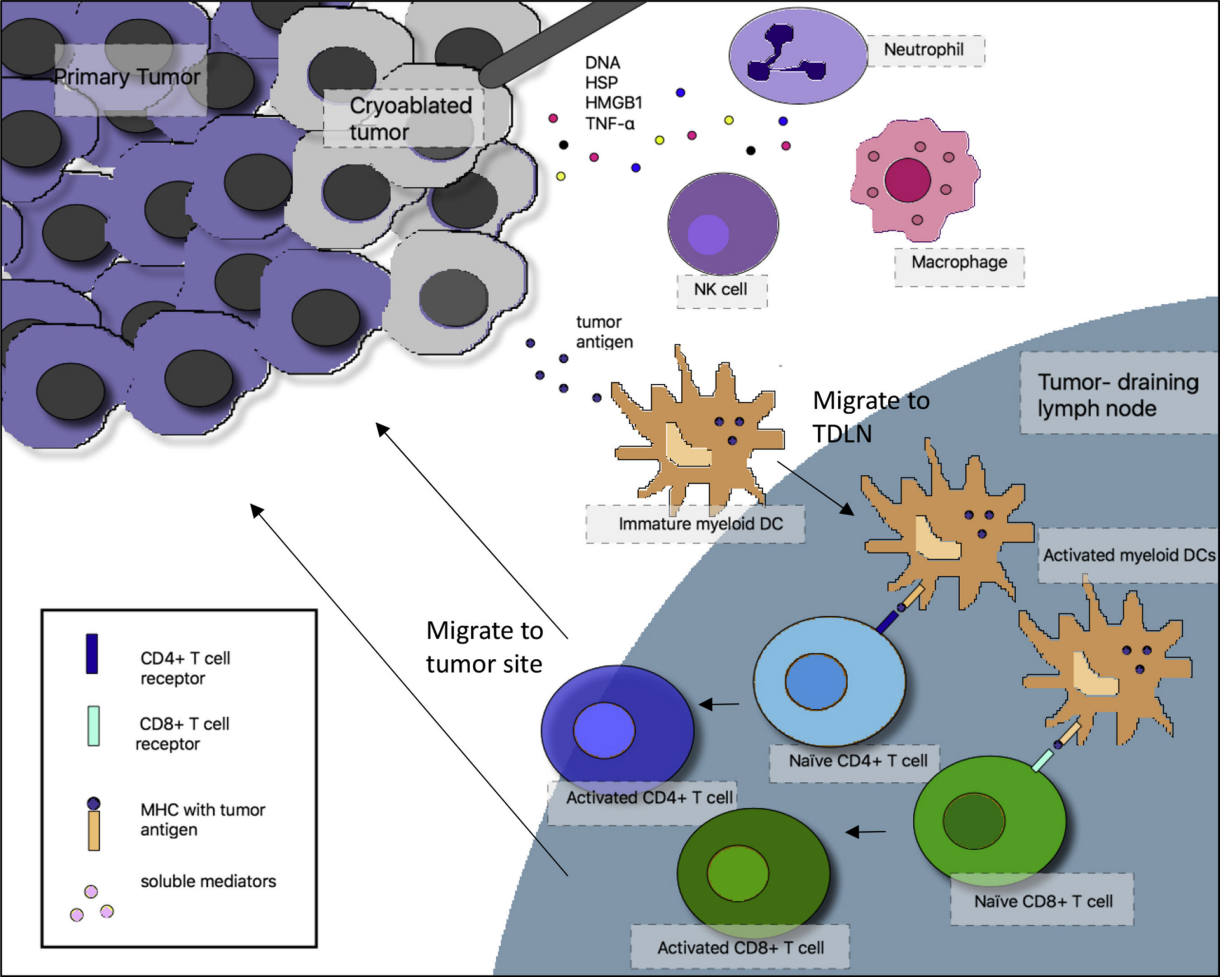
Interactions between the cryoablated tumor and the adaptive and innate immune system. In the proposed mechanism of cryoimmunotherapy, cryoablated tumor cells release tumor antigens, which are taken up by immature myeloid dendritic cells (DCs). In the context of necrotic cell death and generation of inflammatory cytokines after cryoablation, DCs mature and migrate to tumor-draining lymph nodes (TDLN), where they activate tumor-specific T cells, which migrate back to the tumor site. At the same time, dying tumor cells release “danger signals” that recruit macrophages, neutrophils, and natural killer (NK) cells to the tumor site. NK cells can play a direct role in tumor cell lysis. HSP, heat shock protein, HMGB1, high mobility group box 1; MHC, major histocompatibility complex; TNF-α, tumor necrosis factor-α.

Much of the data on immune response to cryoablation are based on pre-clinical models. Briefly, after cryoablation there is an influx of acute inflammatory cells to the cryoablation zone followed by antigen presenting cells. Dying cells release a variety of pro-inflammatory cytokines (e.g., IL-12, IFN-gamma and TNF-alpha) from necrotic tissues at the center of the ablation zone, which serve as “danger signals” to alert the immune system of cellular injury. Antigen presenting cells take up cellular debris including key tumor antigens and then migrate to regional lymph nodes where they interact with T and B-lymphocytes to initiate a cellular and/or humoral immune response (**Figure 1**). It is important to note that cells at the periphery of the ablation zone, where temperatures are not sufficient to induce necrosis, may undergo apoptosis, potentially resulting in release of immune suppressive cytokines (e.g., IL-10, TGF-beta) (7).

Subsequent to cryoablation, anti-tumor antibodies may be formed against ablated tissues, as demonstrated in several pre-clinical cancer models (46–48). It is thought that these antibodies are a marker of an anti-tumor immune response, due to their specificity to tumor tissues. Additionally, murine models demonstrated resistance to re-challenge by the same tumor (but not different tumor lines) after cryoablation which may be due to both humoral- and cell-mediated immunity. Furthermore, the resistance to tumor challenge was demonstrated to be tumor specific (49). Further details of pre-clinical studies of the immunological effects of tumor cryoablation has been covered in detail in previously published manuscripts (7). It is not known how some of these mechanisms may interact with immunotherapies, including immune checkpoint inhibitors.

Notably, not all studies have demonstrated an anti-tumor immune response to cryoablation. In fact, some investigations have suggested an immunosuppressive effect of cryoablation (50–52). Sabel and colleagues proffered that the immune response to cryoablation is dependent upon the cytokine profile triggered by cryoablation, the availability of antigens that can be processed by antigen presenting cells, the mechanism of cell death (specifically necrosis versus apoptosis), and the subsets of phagocytes that process ablated cells (i.e., dendritic cells versus macrophages) (7). Further investigation is needed to understand mechanisms of immune response following cryoablation.

## 7 Studies of cryoablation and immunity in non-small cell lung cancer

Multiple pre-clinical and clinical studies in non-small cell lung cancer have suggested positive outcomes with cryoablation. Several of these studies are have been reported in a previously published review by our group (53). A list of published studies involving cryoablation for the induction of immune response (i.e., cryo-immunotherapy) are included in **Table 1**.

**Table 1:** Published studies of cryo-immunotherapy in lung cancer.

Reference	Population	Ablative technique	Co-administered treatment(s)	Significant findings in combined treatment cohorts versus control and/or pre-treatment cohorts
Gu et al., 2011 (54)	Advanced EGFR- mutated NSCLC	Percutaneous cryoablation	Gefitinib	Increased partial regression (55.6% versus 27.8% with gefitinib alone, p < 0.05). Prolonged PFS (8.4 months versus 5.2 months with gefitinib alone, p < 0.05). Increased 1-year survival (66.7% versus 33.3% with gefitinib alone, p < 0.05)
Yuanying et al., 2013 (55)	Stage IV NSCLC	Percutaneous cryoablation	Intravenous DC-CIK immunotherapy and/or platinum-based chemotherapy	Longer median OS at 20 months versus 10 months (when multimodal treatment protocol included cryoablation versus did not include, p < 0.0001). Longer median OS at 27 months (chemotherapy, cryoablation, and DC-CIK) compared to other treatment groups (p < 0.001). Longer median OS at 18 and 17 months (cryoablation with chemotherapy or with DC-CIK, respectively) versus 8.5 and 12.5 months (chemotherapy alone and chemotherapy with DC-CIK, respectively, p < 0.001)
Lin et al., 2017 (56)	Stage III and IV NSCLC	Percutaneous cryoablation	Intravenous allogenic NK cell immunotherapy	Post-cryoablation peripheral blood total T cell and CD8^+^ T cell populations increased by 1.1-fold each compared to pre cryoablation (p < 0.05). Peripheral blood IL-2 and IFN-γ increased by 2.5 and 3.4-fold, respectively (combined treatment, p < 0.001), and by 1.9 and 2.3-fold (cryoablation alone, p < 0.01), respectively, compared to pre-cryoablation. Improved clinical response rates 3 months post-treatment (63.3% with combined treatment versus 43.3% with cryoablation alone, p < 0.05)
Takaki et al., 2017 (57)	Lung, colorectal, soft tissue, and gynecologic malignancies	Percutaneous cryoablation, MWA or RFA	None	Peripheral blood CTL increased from 27.5% to 30.2% post-ablation (p < 0.03). Peripheral blood CTL/Tregs increased from 18.8% to 21.6% post-ablation (p < 0.05). In 5/19 treated with percutaneous cryoablation, no significant changes in peripheral blood CTL/Tregs or CTL population.
Leppelman et al., 2021 (58)	Stage IV melanoma, NSCLC, various malignancies	Percutaneous cryoablation, SIRT, heat-based ablation, transarterial embolization	Pembrolizumab, nivolumab, atezolizumab	Immunotherapy-related adverse events: 3/7 occurred in cryoablation group (42.8%). Procedural complications greater in the SIRT group compared to cryoablation group (93.3% vs 54.5%)
Feng et al., 2021 (59)	Advanced stage NSCLC	Percutaneous cryoablation	Nivolumab	All adverse effects were manageable and no significant difference was noted between the two groups (*p >* 0.05). Significantly increased number of total T cells, CD8+ T cells and CD4+ T cells in the cryo-nivolumab group (p < 0.05). Significant increase in IL-2 and TNF-beta in cryo-nivolumab group compared to cryoablation group (p < 0.05)

EGFR, epidermal growth factor receptor; NSCLC, non-small cell lung cancer; PFS, progression free survival; DC-CIK, dendritic cell-cytokine induced killer; OS, overall survival; NK, natural killer; RFA, radiofrequency ablation; MWA, microwave ablation; CTL, cytotoxic T lymphocyte; Treg, regulatory T cell; SIRT, selective internal radiation therapy; SCLC, small-cell lung cancer; ORR, objective response rate; DCR, disease control rate.

Additionally, more recently Xu et al. studied the clinical outcomes and immune response of patients with lung cancer who received systemic therapies with or without bronchoscopic cryoablation for *endobronchial* disease (60). Bronchoscopic cryotherapy was applied *via* cryoprobe *via* a single freeze-thaw cycle lasting 3 to 4 minutes. Improved response rate was noted in the cryoablation arm and there was suggestion of decreased serum tumor markers (e.g., carcinoembryonic antigen) and changes in CD4 and CD8 T-cell subsets in the peripheral blood following cryotherapy. It is important to note that the methodology in this study was limited by absence of standardization of systemic therapy, and that patients in this study received systemic chemotherapy or molecular targeted therapy rather than immunotherapy. However, the concept of combining cryoablation, in this study delivered bronchoscopically, with systemic therapies – particularly immune checkpoint inhibitors – is an approach that warrants further investigation.

## 8 Cryoablation in combination with immune-checkpoint inhibitors

**Figure 2:**
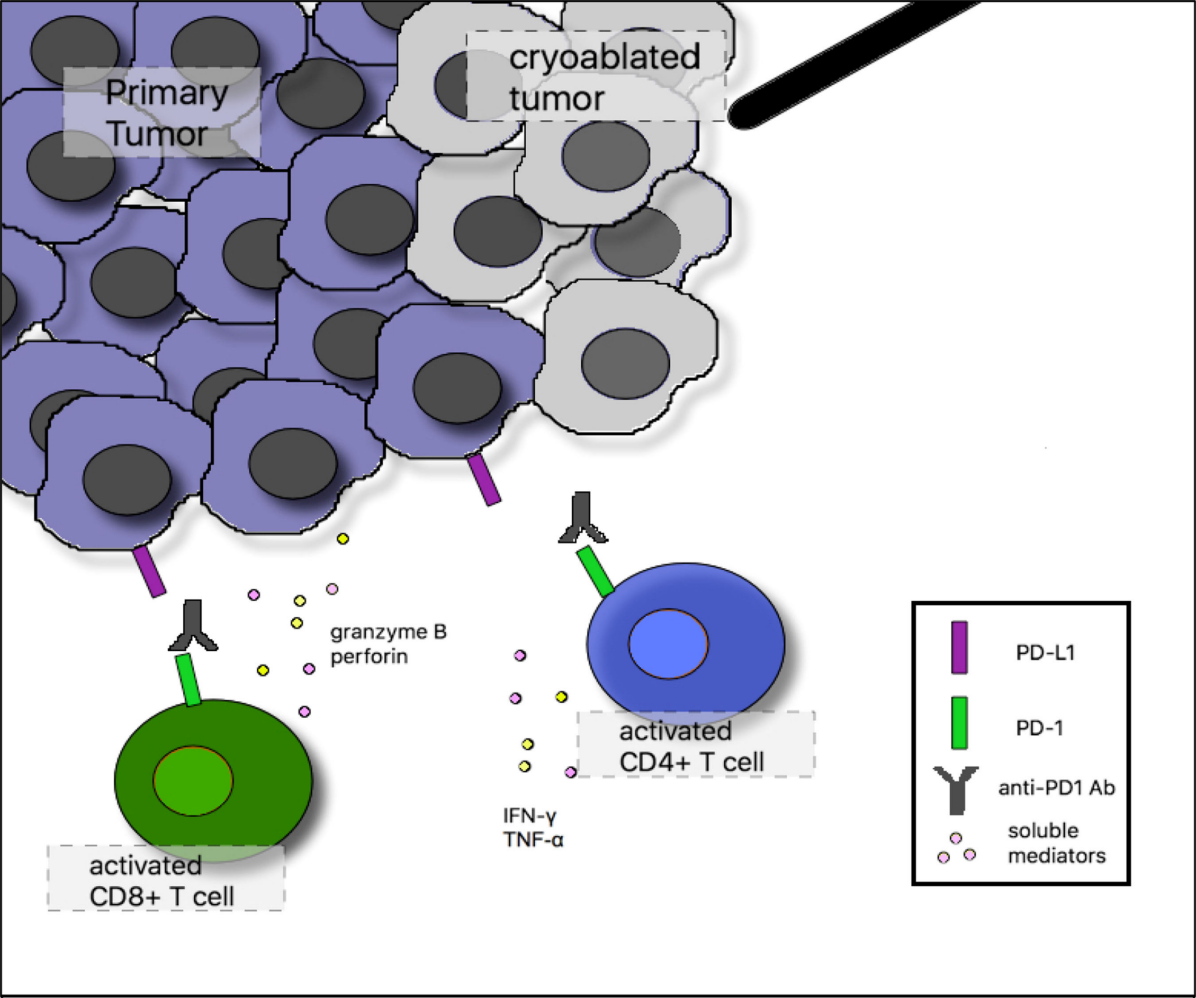
Proposed mechanism of synergy between cryoablation and anti–programmed cell death 1 (PD-1) monoclonal antibodies. In the tumor microenvironment after cryoablation, activated CD4-positive T cells augment immune responses through release of the proinflammatory cytokines interferon gamma (IFN-γ) and tumor necrosis factor-α (TNF-α). Activated CD8-positive T cells induce tumor cell lysis through release of granzyme B and perforin. Tumor cells can inhibit infiltrating tumor-specific T cells by up-regulating programmed death ligand 1 (PD-L1) surface expression, which binds PD-1 on the surface of activated T cells. Anti–PD-1 monoclonal antibodies block this inhibitory interaction and sustain T-cell activation.

There are theoretical benefits of combining cryoablation with immune checkpoint inhibitors, as they work on different elements of anti-tumor immunity. Based on a proposed mechanism, releasing tumor antigens while the immune system is under the influence of immune checkpoint blockade may prevent T-cell exhaustion or anergy and reinstate anti-tumor immunity (**Figure 2**). At least one case report has demonstrated a robust clinical response due to the combination of bronchoscopic cryoablation and camrelizumab, a humanized monoclonal antibody against programmed cell death protein 1 (PD-1), in a patient with advanced sarcomatoid carcinoma of the lung (32).

Leppelman et al. performed a single-center retrospective cohort study evaluating the safety of locoregional therapies in patients receiving systemic immune checkpoint inhibitors for several malignancies including non-small cell lung cancer (12 of 65 patients) (58). Notably, locoregional therapies included thermal ablation, embolization, or selective internal radiation therapy, with cryoablation accounting for 28% of procedures. Immune-related adverse events occurred in 10.7% of patients, all less than or equal to grade 3, with none occurring in those with non-small cell lung cancer. It is important to recognize the potential for adverse effects from combining systemic and locoregional therapies. At least in this experience, there were no safety signals for combination therapies noted with cryoablation.

Feng et al. investigated the safety and efficacy of percutaneous cryoablation with or without nivolumab in patients with stage IIIB or IV non-small cell lung cancer (59). The combination of cryoablation with nivolumab versus nivolumab alone, was associated with an increase in the number of immune effector cells such as total CD4+ and CD8+ T cells and natural killer (NK) cells and the serum levels of inflammatory cytokines (i.e., IL-2, TNF-beta, IFN-gamma) in the cryotherapy plus nivolumab group. Adverse events were all less than or equal to grade 3 and without significant differences among the two groups. The combination of nivolumab with cryoablation resulted in significantly reduced circulating tumor cells and decreases in the tumor markers CYFRA21-1 and neuron-specific enolase (NSE). This study was limited by its retrospective nature and unconventional treatment strategies.

Further data regarding the safety and efficacy of the combination of cryoablation with immune-checkpoint inhibitors is needed. Several studies are currently investigating cryoablation in combination with systemic immunotherapies in non-small cell lung cancer (see **Table 2**). Additionally, studies which characterize both the tumor microenvironment and peripheral immune cells before and after cryoablation will be crucial to further understanding potential mechanisms of synergy.

**Table 2:** Table 2. Ongoing clinical trials of cryo-immunotherapy in lung cancer.

Ablative technique	Cancer	Co-administered therapy	Primary end point	Secondary end point	Planned end date	Status	Location	Clinical trial identifier
		Immunotherapy						
Percutaneous cryoablation	Multiple primary lung cancers	Camrelizumab and apatinib	Safety	Response rate	12/2021	Unknown	Guangzhou Institute of Respiratory Disease	NCT04201990
Percutaneous cryoablation	Metastatic NSCLC	Pembrolizumab, pemetrexed, carboplatin	1-year OS	Response rate	8/2023	Recruiting	Institut Bergonié	NCT04339218
Percutaneous cryoablation	Metastatic lung cancer	Various immune checkpoint inhibitors	Safety and feasibility	Response rate	1/2022	Recruiting	Massachusetts General Hospital	NCT03290677
Percutaneous cryoablation	Advanced NSCLC	None	Safety and feasibility	Antitumor immune response	6/2023	Recruiting	NYU Langone Health	NCT04049474
		EGFR TKI						
Percutaneous cryoablation	Advanced NSCLC with EGFR mutation	Icotinib	PFS	OS	8/2017	Enrollement completed	Fuda Cancer Hospital, Guangzhou	NCT02744664

NSCLC: non-small cell lung cancer; EGFR TKI: epidermal growth factor receptor tyrosine kinase inhibitor; PFS: progression-free survival; OS: overall survival

## 9 Discussion

Historically, cryoablation has been a means of local tumor control in patients who are unfit for surgery. However, there is growing interest in cryoablation as a means to induce an immunologic effect which is specific to the ablated tumor. With recent development of immunotherapies for lung cancer, cryoablation has entered a new era, with the potential to synergize with systemic checkpoint inhibitors. Several important questions arise regarding the role of cryoablation in non-small cell lung cancer based on this review of recent literature.

First, what is the optimal ablative dose and number of lesions to target? Despite increasing knowledge and experience with various cryoablation protocols, knowledge of the best strategy remains limited. As multiple studies have suggested that immune response is variable after cryoablation (61, 62), determining the most effective dose for immune stimulation (which may be different from achieving maximal cell death) is of utmost importance.

Second, is there any role for cryoablation of tumor-draining lymph nodes? Although at present in limited capacity, some have postulated nodal cryoablation as a means to combat immune checkpoint inhibitor resistance. Thoracic lymph nodes in non-small cell lung cancer may be readily accessible for EBUS-mediated bronchoscopic cryoablation, and may be therefore present an opportunity for future investigation into this topic.

Additionally, is there any potential neoadjuvant role of cryoablation and immunotherapy prior to resection of early stage non-small cell lung cancer? Impressive pathological responses have been observed with neoadjuvant checkpoint inhibitors prior to resection (63), and the addition of cryoablation to such a regimen at the time of bronchoscopy could theoretically further enhance this effect, particularly in those lung cancers with “cold” baseline immune microenvironments (low PD-L1 expression and absence of infiltrating T cells).

Accumulating evidence suggests that cryoablation may generate an anti-tumor immune response at the cellular level by potentiating the cancer-immunity cycle, especially when used in combination with other immunotherapies. At the clinical level, limited data suggest a potential improvement in disease regression and progression-free survival when combination of therapy and cryoablation is used. More clinical studies are needed to further determine how cryoimmunotherapy translates to clinical outcomes.

**Figure 3:**
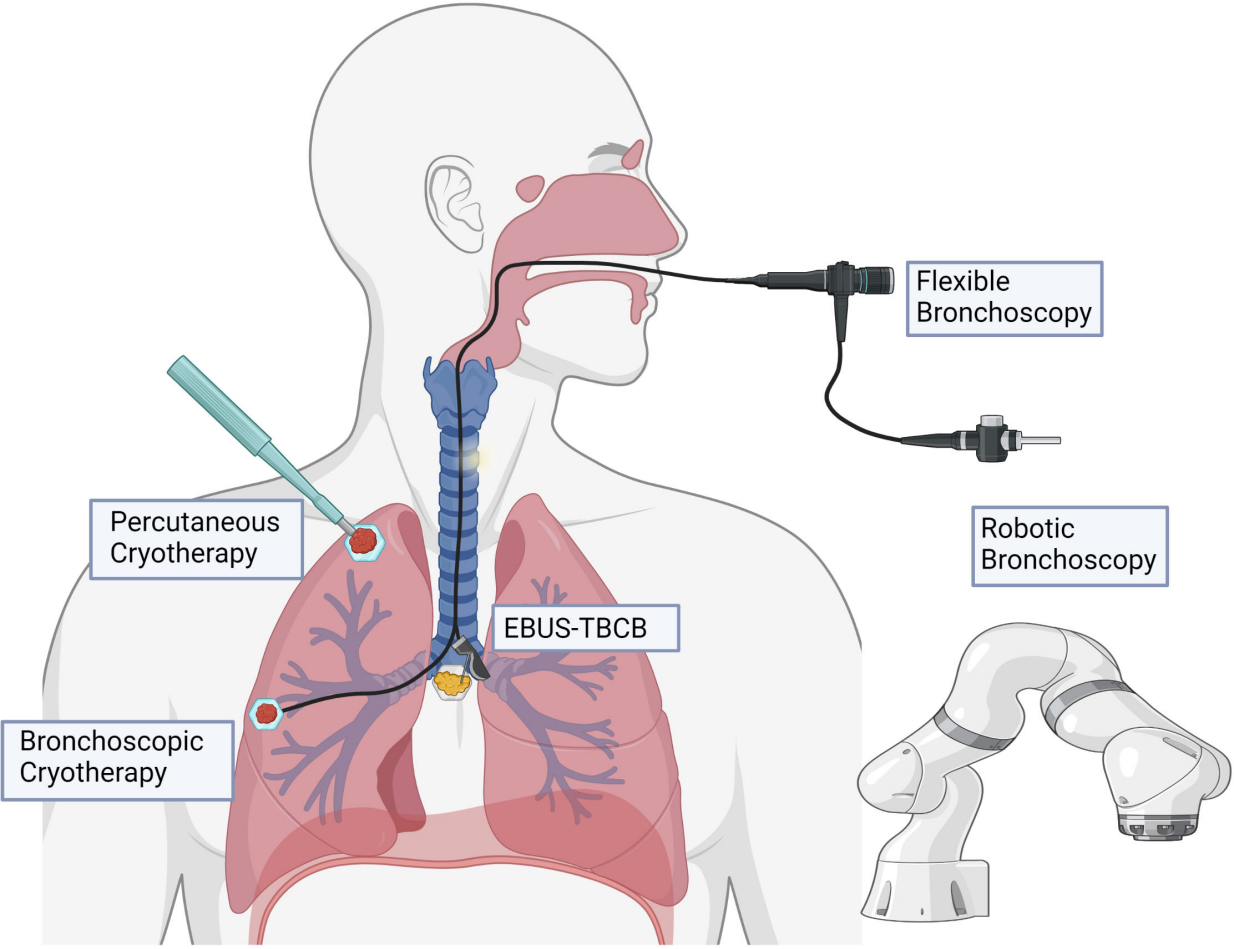
Potential modalities for delivery of cryotherapy. Percutaneous cryoablation is typically delivered with image guidance (e.g., computed tomography). Bronchoscopic cryotherapy can be delivered to endoluminal lesions in proximal and segmental airways under direct visualization, as well as to more peripheral lesions with the assistance of robotic bronchoscopy. Endobronchial ultrasound guidance may allow for potential to target tumor-draining lymph nodes in the mediastinum.

Due to technical limitations, cryoablation has largely been performed *via* percutaneous approach, although recent progress in the field of interventional pulmonology may allow for routine bronchoscopic cryoablation in the near future (**Figure 3**). Additionally, further research into discovering optimal dose and mechanisms of cryoablation will be essential to maximize this potential therapy.

## Author contributions

AV, AD, and DS designed this study. AV performed the initial literature search and wrote the first draft of the manuscript. AD and DS revised the manuscript and provided additional sources. All authors contributed to the article and approved the submitted version.
